# Soil microbial community shifts with long-term of different straw return in wheat-corn rotation system

**DOI:** 10.1038/s41598-020-63409-6

**Published:** 2020-04-14

**Authors:** Yao Su, Man Yu, Hui Xi, Jinling Lv, Zhenghua Ma, Changlin Kou, Alin Shen

**Affiliations:** 1 Institute of Environment, Resource, Soil and Fertilizer, Zhejiang Academy of Agricultural Sciences, Hangzhou, 310021 China; 2 Institute of Plant nutrient, Environment and Resource, Henan Academy of Agricultural Sciences, Zhengzhou, 450002 China

**Keywords:** Applied microbiology, Microbial ecology

## Abstract

Despite the integral role of the soil microbial community in straw decomposition, we still have a limited understanding of the complex response of microbial community to long-term of crop straw return in rotation system. Here we report on the structural and functional response of the soil bacterial and fungal community to more than 10 years of straw return in wheat-corn rotation system. Compared with single-season straw return, soil microbial phosphor lipid fatty acids (PLFAs) and catabolic activity were improved more greatly with double-season straw return. The relative abundance of bacteria and fungi decreased with double-season straw return, but increased with single-season straw return. The copiotrophic bacteria were more represented in the soils with corn straw return, while oligotrophic groups were more represented in soils with wheat straw return. Compared with wheat straw return, lower fungal community diversity and higher abundance of fungal pathogen (identified to be *Leptosphaeria*) were observed with corn straw return, especially at high return rates. Redundancy analysis showed that soil available potassium (*P* = 0.008) and ratio of C to N (*P* = 0.048) significantly affected the soil bacterial community, while soil electric conductivity (*P* = 0.04) was the significant factor impacting soil fungal community. It suggests that full corn straw return might have positive impact on soil mineral nutrient but negative impact on soil fungal community diversity and pathogenic risk, mainly due to the change in soil electric conductivity.

## Introduction

Crop straw returning has been demonstrated to have positive impact on soil aggregation, structure, water-holding capacity and soil nutrient availability^1–4^. Incorporating crop straw into soils is a prevailing agricultural practice in China, but excessive straw return always brings challenges for crop production and soil quality, such as hampering root penetration of the next crop^5^, reducing soil nitrogen (N) level^6–8^, and contributing to disease and weed problems^5,9^, particularly in the single year rotation system. Returning crop straws to soils at relatively higher rates may provide favorable conditions for potential pathogenic population growth, and then contributed consequently to disease, such as maize root-rot^10^, wheat common rot and sharp eyespot^11^.

In the plains of northern China, double-cropping system of winter wheat and summer corn takes up 60% of the arable land, which is one of the largest regions of agricultural production in China^12,13^. In this rotation system, corn is seeded in early June, after the winter wheat harvest and harvested in mid-September; winter wheat is then seeded in early October and harvested in the following June. Currently, full straw return is applied widespread in this region and it brings about problems for seed sawn, soil-borne disease and crop production^11^. Compared with single-season straw return, double-season of full straw return increased soil organic matter, available phosphorus (AP) and potassium (AK), but decreasing alkaline-hydrolyzed N (AN), significantly^6,7^. Thus, more reasonable straw returning mode is required.

Soil microbial communities play key roles in not only straw decomposition^14^ but also soil nutrient cycling and the maintenance of soil health^15,16^. Changes in soil physiochemical characteristics, due to the difference of straw quality, straw return rates and climate, had considerable impact on the soil microbial communities^14,17–19^. Corn straw return increased the fungal biomass, but did not affect the bacterial in long-term (>30 years) in North-central China, especially at relatively high return rates (>4500 kg ha^−1^)^19,20^. Chen *et al*.^20^ reported that the biomass and composition of soil microbial community responded differently to straw return in different sites with short-term rice and wheat straw return. The catabolic diversity and activity was also improved by straw (rice, wheat and corn straw) return in short (three years) and long term (30 years)^19,20^. However, most of these studies applied phosphorlipid fatty acid (PLFA) analysis and Biolog plate method, which were limited in obtaining accurate information about the taxonomic composition and carbon metabolism of soil microbial community. Thus, it is difficult to understand the correlation between the change of soil microbial community and the risk of soil health and further the crop safety. Such correlation can help to better understand the effect of straw return on soil health and its microbial mechanism.

Thus, in this study, a field experiment with different rates of straw return was conducted under wheat-corn rotation system in North China Plain for 11 years. To investigate the response of soil microbial community structure and taxonomic composition, PLFA analysis and Miseq sequencing technique were both applied. MicroResp measurement was conducted to evaluate the soil catabolic activity. It is hypothesized that (1) different rates of straw return might shift the soil microbial community structure and affect the activity differently; (2) high return rates might increase the pathogenic risk for crop, by increasing the abundance of pathogenic microbes in soils.

## Materials and methods

### Site description and experimental setup

The field experiment was established in the year of 2005 at the Junxian Farming Experimental Station, Henan province, China (35.67N, 114.54E). This site has a warm temperate, sub-humid continental monsoon climate. The annual average temperature is 12–16 °C, with the highest of 24–29 °C in July and the lowest of −3-3 °C in January.

The experiment was conducted on a winter wheat-summer corn rotation system using a split-plot design with three replicates. No-fertilizer and no-straw return was set as control, labeled as Blank. Double-season straw return at rates of 0, 4900~5500 (equal to about 50% of crop yield in each single season) and 9800~11000 kg ha^−1^ (equal to about 100% of crop yield in each single season) under combined N, P and K fertilization at 450, 210 and 240 kg ha^−1^ yr^−1^, respectively, was labeled as CW-0, CW-50% and CW-100%, respectively (corn straw labeled as C, wheat straw labeled as W, number referred to return rate). Only corn straw return at rates of ~5500 and ~11000 kg ha^−1^ were labeled as C-50% and C-100%, respectively, while only wheat straw return at rates of ~4900 and ~9800 kg ha^−1^ were labeled as W-50% and W-100%, respectively. Before the next season-crop sowing, all straws (except crop stubble) were crushed into pieces and directly returned to the soils, and then the soil was turned over. Other field management practices followed standard farming practices.

### Soil sampling and analysis

The soil was of the fluvo-aquic type, with the content of soil organic carbon (SOC) of 9.98 g kg^−1^, total N (TN) of 0.92 g kg^−1^, and with AN, AK and AP content of 44.18, 109.45 and 6.49 mg kg^−1^, respectively. The method of soil sampling and pre-treatment was consistent with those described as Su *et al*.^21^. Briefly, after wheat harvested in June 2016, three soil cores (0–20 cm depth, 2 cm in diameter) were collected in each plot, and then sieved to pass through a 2-mm mesh, after which they were immediately analyzed for microbial catabolic capacity. Additionally, a part of the subsamples were stored at −70 °C for microbial PLFA and DNA extraction, while the other subsamples were used for determination of soil pH, electric conductivity (EC), SOC, TN, AN, AK and AP content.

### Soil nutrient characteristics

Soil pH was measured using the electrode method (5:1 water/soil ratio), and soil EC was measured with a conductivity meter (2.5:1 water/soil ratio)^21^. SOC was measured using the potassium dichromate volumetric method with external heating^22^. AN was measured by alkaline hydrolysis^23^. AP was measured by the Mo-Sb colorimetric method^24^. AK was measured by flame photometry after NH_4_OAc extraction^25^. Fresh soil samples were oven dried at 105 °C to determine soil moisture^26^.

### Microbial catabolic capacity using MicroResp method

The MicroResp method was conducted to determine the ability of the soil microbial community to metabolize a range of carbon sources by using the MicroResp Starter Kit (The James Hutton Limited, Craigiebuckler, Aberdeen, U.K.). The method was followed as described by Campbell *et al*.^27,28^. Briefly, about 0.5 g of sieved soil samples was placed in a 96-deep-well micro-plate by filling 96 holes of container with soil. The weight of the microplate plus soil was recorded and subtracted from the microplate tare weight. Water was added to each soil sample to reach a water potential of 0.01 MPa (pF2).

Each well received separate organic substrates which were carbohydrates (D-glucose, L-arabinose, D-fructose, trehalose and D-galactose), carboxylic acids (citric acid, oxalic acid, malic acid and g-aminobutyric acid (GABA)), phenolic acid (3,4-OHbenzoic acid) and amino acid (L-cysteine, L-alanine, L-lysine, arginine and n-acetyl glucosamine (NAGA)). These carbon sources are ecologically relevant as: components typically found in or added to soils such as crop residues, root exudates and as sources of mineralized nutrients^28^. Water was added as control. The catabolic profiling was carried out with triplicates per substrate per soil sample. The deep-well micro-plates were sealed individually to a colorimetric CO_2_-trap microplate and incubated in the dark at 29 °C for 6 h^27^. CO_2_-trap absorbance was measured at 570 nm with a Victor3 multilabel counter (PerkinElmer) immediately before and after 6 h incubation. A calibration curve of absorbance versus headspace equilibrium CO_2_ concentration was fitted to regression model^27^.

### PLFA and DNA sequencing analysis

Triplicate subsamples of ~4.0 g freeze-dried soil from each treatment were used to extract PLFA using the method as described by Yu *et al*.^29^. The phospholipid ester-linked fatty acid methyl esters were dissolved in ethanol for further GC-MS analysis as described by Yu *et al*.^29^. The GC-MS system was TRACE 2000, Palaris Q CP-Sil 5CB Low Bleed/MS with fused-silica column (30 m × 0.25 mm × 0.25 mm). The injection port temperature was set to 250 °C. The oven temperature was programmed from 60 to 230 °C, increasing at rate of 58 °C min^−1^ and held isothermally at 230 °C for 20 min. The detector temperature was set at 200 °C^29^.

DNA extraction was performed on each composite soil sample using Power Soil DNA Isolation Kit (MOBIO Laboratories, Inc., NewYork, USA) according to the instruction manual. The extracted DNA was quantified using the Nanodrop ND-1000 spectrophotometer. The extracted DNA was analyzed by Illumina MiSeq sequencing. PCR amplification was carried out using the primer set of 515F and 907R for the V4-V5 region of bacterial 16S rRNA gene, and the primer set of ITS1 and ITS2 for the ITS region of fungi. The PCR reaction system and conditions, the method of purifying amplicons and selecting high-quality raw reads were followed as Su et al. described^21^. The taxonomy of each 16S rRNA and ITS gene sequences were analyzed by RDP Classifier algorithm (http://rdp.cme.msu.edu/) against the database of SILVA128 for 16S rRNA gene and UNITE7.0 for ITS using confidence threshold of 70%.

### Statistical analysis

Significant differences and Pearson’s linear correlation analysis was analyzed in the SPSS software program version 19.0. Redundancy analysis (RDA) was performed with the package CANOCO 4.5. The Monte Carlo permutation test based on 9999 random permutations was used to test the statistical significance of the relationship between the variations of the microbial community with the soil physiochemical properties.

## Results

### Soil nutrient characteristics

**Figure 1:**
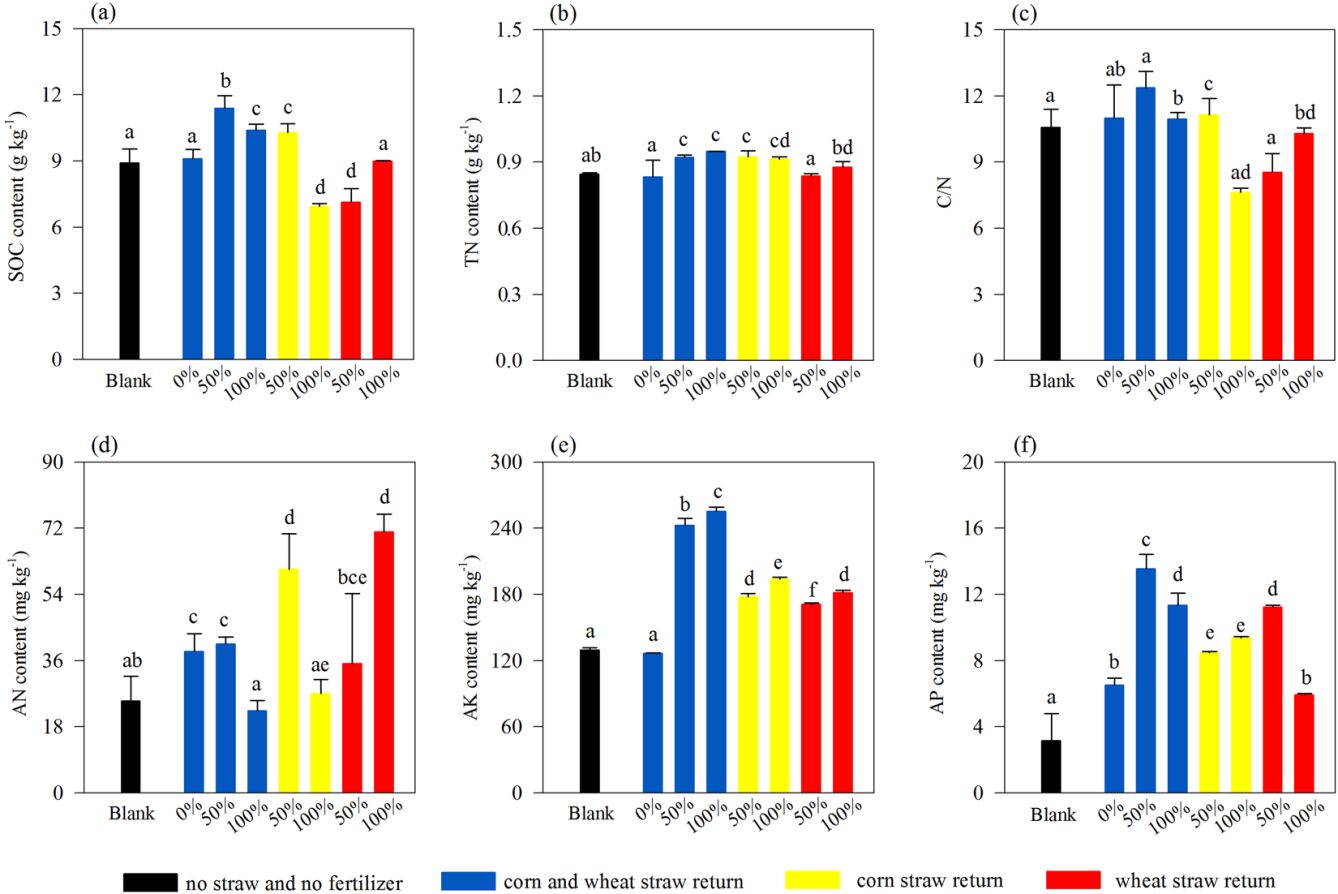
General description of soil nutrient characteristics, including the content of SOC (**a**), TN (**b**), ratio of C to N (**c**) and the content of AN (**d**), AK (**fe**) and AP (**gf**), respectively. Different letters indicate significant difference *(P* < 0.05) analyzed by using ANOVA.

The soil nutrient content differed among the treatments significantly (Fig. 1 and Table S1). Compared with CW-0, significantly higher SOC was observed in CW-50%, CW-100% and C-50%, with increase of 25.2%, 14.2% and 13.0%, respectively, while with the decrease of 23.6%, 21.6% and 1.0% in C-100%, W-50% and W-100%, respectively. The relatively higher TN was also observed in CW-50%, CW-100% and C-50%, with the increase of 10.6%, 13.8% and 10.9%, respectively. AN increased significantly in C-50% and W-100% when compared with CW-0. The content of AK increased from 126.5 mg kg^−1^ in CW-0, to 171.0–255.5 mg kg^−1^ in the soils with straw return, especially in double-season straw return (CW-50% and CW-100%). The content of AP was also relatively higher in the soils with straw return than that in CW-0, except for W-100%. In addition, the soil EC was greatly lower in the soils with only wheat straw return (198.2 and 194.4 μs cm^−1^) than that in the soils with only corn straw return (200.0 and 200.2 μs cm^−1^) and double-season straw return (204.0 and 218 μs cm^−1^) (Table S1).

### Soil microbial community structure based on the PLFA analysis

**Figure 2:**
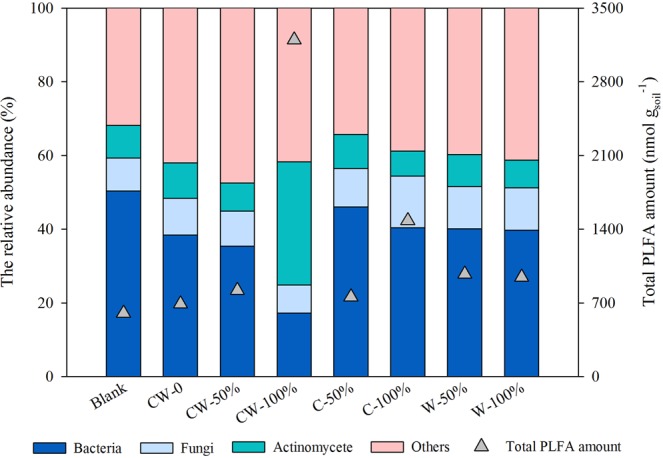
Abundance of bacteria, fungi and actinomycete PLFA in the soils with different straw return treatments.

The amounts of total PLFAs increased greatly in the soils with straw return, with the PLFA amounts of 756.7–3197.1 nmol g_soil_^−1^, which was 1.1–5.3 times of that in Blank and CW-0 (Fig. 2). Compared with CW-0, the relative abundance of bacteria and fungi both decreased in the treatments with double-season straw return (CW-50% and CW-100%), while it increased with single-season straw return. Interestedly, the relative abundance of actinomycete increased greatly in CW-100%, which was about 2.8–4.9 times of that in other treatments. These results indicated that the soil microbial community structure had different responses to single- and double-season straw return.

### Effect of straw return on soil microbial community *alpha*-diversity

Miseq sequencing was applied to analyze both bacterial and fungal community in all soil samples. About 33922 ± 6534 bacterial 16S_V4-V5_ and 64118 ± 57301 fungal ITS1-2 high-quality sequences per sample was obtained for the tested DNA samples. Cluster yielded 2186 ± 102 and 143 ± 57 OTUs per sample for bacterial and fungal sequences, respectively.

A*lpha*-diversity indices (Shannon and Simpson index) of soil bacterial and fungal community were calculated as shown in Table 1. Compared with fungal community, bacterial community had higher richness and evenness in all treatments. There was no significant difference on the bacterial community diversity among treatments with straw return, but the remarkable impact was observed on the fungal community. Compared with Blank, the richness and evenness of fungal community of the soils under compound fertilization all reduced significantly, except for W-50% and W-100%. Compared with the double season straw return, lower Shannon index and higher Simpson index were observed in soils with single-season of corn straw return. The results indicated that wheat straw return improved soil fungal community diversity, but more specific fungi might be selected as the dominant by corn straw return, causing the reduction of fungal community diversity.

**Table 1:** *Alpha*-diversity indexes for soil samplies with different straw return treatments.

Treatment	Shannon index		Simpson index	
	Bacterial community	Fungal community	Bacterial community	Fungal community
Blank	6.40^a^	3.12^b^	0.004^a^	0.101^b^
CW-0	6.44^a^	1.21^c^	0.004^a^	0.398^c^
CW-50%	6.32^a^	1.33^c^	0.005^a^	0.351^c^
CW-100%	6.46^a^	1.31^c^	0.004^a^	0.317^c^
C-50%	6.33^a^	1.10^d^	0.005^a^	0.431^d^
C-100%	6.43^a^	1.13^d^	0.004^a^	0.419^d^
W-50%	6.52^a^	3.61^e^	0.004^a^	0.048^e^
W-100%	6.33^a^	3.37^b^	0.005^a^	0.074^b^

Note: different lower case letters in the table indicate significant difference at the level of *P* < 0.05.

### Response of microbial community composition to straw return

**Figure 3:**
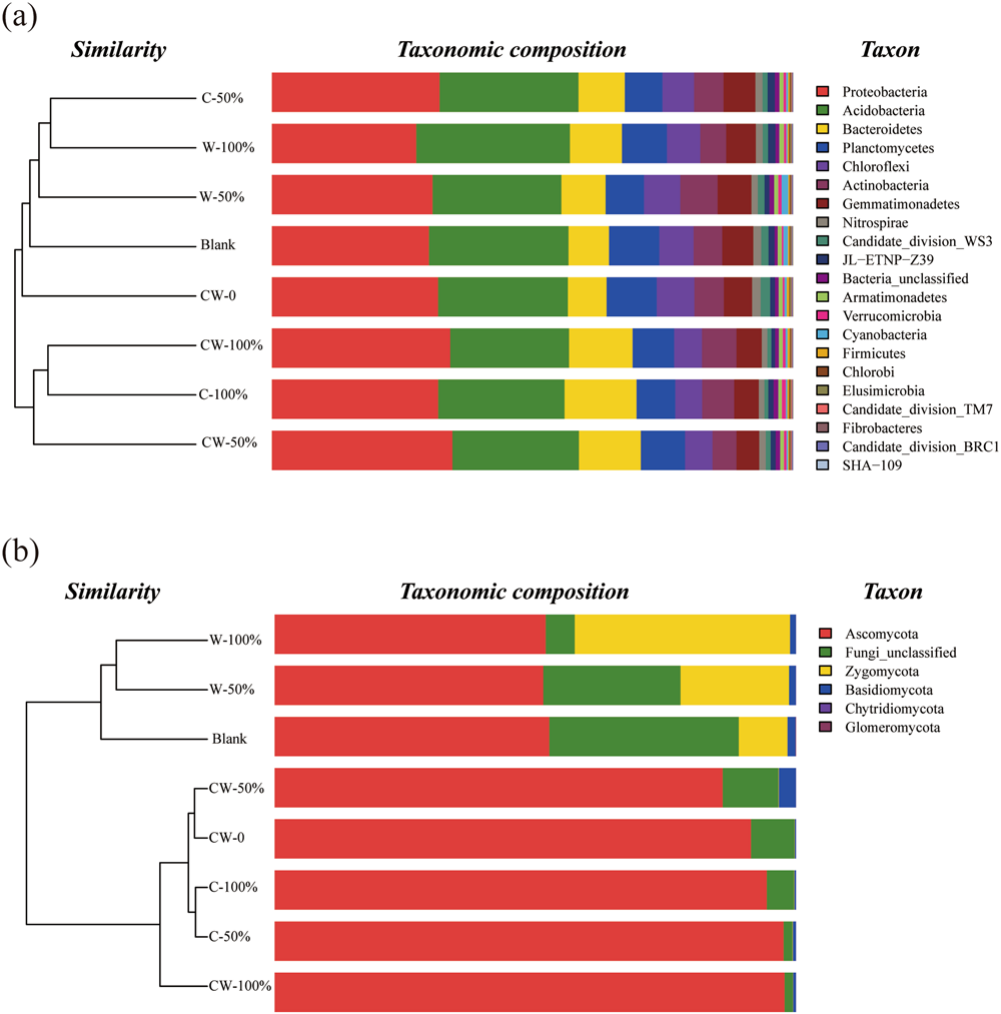
The relative abundance of dominant phylum of bacterial (**a**) and fungal (**b**) community in the soils with different straw return treatments.

Proterobacteria, Acidobacteria, Bacteroidetes and Planctomycetes were the dominant bacterial phylum, accounting for 27.7–34.6%, 22.8–29.5%, 7.4–13.8% and 7.2–9.6% of all sequencing reads, respectively (Fig. 3a). Some other bacteria including Chloroflexi, Gemmatimonadetes, Actinobacteria and Nitrospirae were also identified in all the soils, accounting for about 1.1–7.2% of all sequencing reads. Compared with CW-0, the relative abundance of Bacteroidetes increased by 1.1–6.4% in the soils with straw return, especially at high return rates and with corn straw return. On opposite, the relative abundance of Chloroflexi and Nitrospirae decreased by 0.3–2.1% and 0.3–0.6%, respectively in the soils with straw return, especially in the soils with double-season straw return and only corn straw return. In addition, the relative abundance of Gemmatimonadetes decreased from 5.4% in CW-0 to about 4.3%, 4.8% and 4.7% in CW-50%, CW-100% and C-100%, respectively, while it increased to 5.7% and 6.5% in W-50% and W-100%, respectively. Cluster analysis showed that CW-50%, C-100% and CW-100% clustered together, while the other treatments clustered together. It indicated that the soil bacterial community had similar response to only corn straw return and the double-season straw return, which was different from the treatments with only wheat straw return.

**Figure 4:**
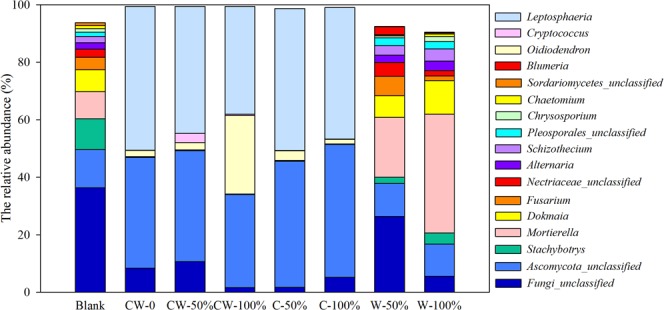
The relative abundance of dominant genus of fungal community in the soils with different straw return treatments.

Regarding to the fungal community, Ascomycota, Zygomycota, Basidiomycota, Chytridiomycota and Glomeromycota were detected in all soil samples, and Ascomycota was the dominant, accounting for 51.5–97.8% of all sequencing reads (Fig. 3b). The relative abundance of Ascomycota increased significantly from 54.2% in Blank to about 85.9–97.8% in the soils with double-season straw return and single-season of corn straw return, while the relative abundance of the other fungi reduced greatly in these soil samples. The dominant fungi belonging to Ascomycota in these soil samples were detected as genus of *Leptosphaeria*, with the relative abundance increasing from 0.0% in Blank to 44.1%, 37.4%, 49.3% and 45.7% in CW-50%, CW-100%, C-50% and C-100%, respectively (Fig. 4). But the relative abundance of other pathogenic fungi, *Alternaria* and *Fusarium*, reduced from 2.2% and 4.3% in Blank to nearly 0.0% in these soils. In addition, the relative abundance of *Oidiodendron* increased from 0 in Blank to 1.5%~27.3% in these soils. On the contrary, in the soils with only wheat straw return (W-50% and W-100%), the relative abundance of Ascomycota was similar to that in Blank, accounting for 51.5% and 52.0% of total sequencing reads, respectively, while the relative abundance of Zygomycota increased remarkably from 9.4% in Blank to 20.8% and 41.3% in W-50% and W-100%, respectively. This global increase was all due to the stimulation of the genus of *Mortierella*. Cluster analysis showed Blank treatment and the treatments with only wheat straw return were clustered together, while other treatments were clustered together. It indicated that returning corn straw shifted soil fungal community structure greater than wheat straw.

### Effects of straw return on microbial catabolic capacity

**Figure 5:**
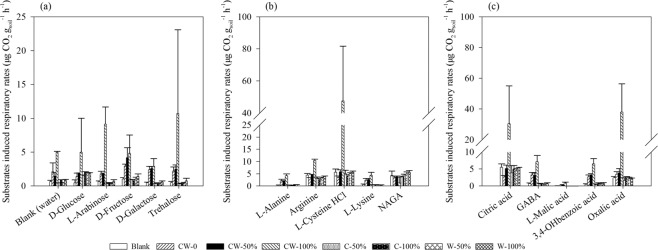
Catabolic profiling obtained with MicroResp™ assay in response to the different treatments. Carbon sources included carbohydrates (**a**), amino acids (**b**) and organic acids (**c**). Data are mean values + SD (n = 3).

The respiration of the soil microbial communities induced by different organic matter can be used as an indicator to assess their functional capacity in organic matter decomposition and nutrients mineralization. As shown in Fig. 5, much higher substrates induced respiration rates were found in CW-100% and CW-50%, with the average rate of 11.8 and 3.1 μg C-CO_2_ g_soil_^−1^ h^−1^, respectively, while the average rate of other treatments was all less than 2.5 μg C-CO_2_ g_soil_^−1^ h^−1^. The higher respiratory rates of D-glucose were presented in soils with straw return (1.6–5.0 μg C-CO_2_ g_soil_^−1^ h^−1^) than Blank and CW-0, with the rate of 0.5 and 1.4 μg C-CO_2_ g_soil_^−1^ h^−1^, respectively. The respiratory rate induced by L-cysteine, Citric acid and Oxalic acid were greatly higher than other substrates in all the soils (*P* < 0.05), and the highest rate was observed in CW-100%, about 47.5, 30.4 and 38.0 μg C-CO_2_ g_soil_^−1^ h^−1^, respectively. Moreover, the highest respiratory rate induced by Trehalose was also observed in CW-100% (10.7 μg C-CO_2_ g_soil_^−1^ h^−1^), which was about 4.9–34.6 times of others.

## Discussion

### Long-term straw return drives soil microbial community change

The total PLFAs were significantly more abundant in soils receiving straw return, mainly for that these soils had higher contents of SOC and TN. It reported that higher contents of SOC and TN can provide sufficient nutrients for more soil microorganisms^30^. The increase of the abundance of total PLFA, fungi, bacteria and actinomycete were all observed in previous studies in short or long-term straw return^19,20^. Interestingly, single and double season straw return had different effect on soil microbial community structure. Compared with no straw return (CW-0), the relative abundance of bacteria and fungi decreased in the soils with double-season straw return, especially at high rates, but increased slightly in the soils with single-season straw return. This might be associated with soil nutrient contents and the pathogenic risk. Compared with single-season straw return, relatively higher content of AP but lower content of AN was observed in double-season straw return in this study. It was consistent with the results reported by Shan *et al*.^2^ that more organic acids released during straw decomposition and activating more organic P, leading to an increase in soil P availability. But when straws provided sufficient C and N source for microbial growth, the microbes might consume more soil native organic matter and available N to synthesize the cellular structures, and thus the mineralization of soil organic matter should be accelerated, and finally cause the reduction of SOC and available N^6,7^, such as in the treatment of CW-100% and C-100%. In addition, it reported that actinomycetes have varying degrees of inhibitory activity against pathogenic fungi to ameliorate the pathogenic effect^31,32^. Thus, the increase of actinomycetes in double season straw return might be associated with the relatively higher abundance of fungal pathogen, such as the genus of *Leptosphaeria* detected in the experimental soils^33^.

The response of soil bacterial and fungal community structure to only corn straw return was similar to double-season straw return, but different from the treatment with only wheat straw return. The copiotrophic bacterial, such as *Bacteroidetes* increased in the soils with straw return^34^, and it were more represented in the soils with double-season straw return and with only corn straw return than in the soils with only wheat straw return, where oligotrophic groups such as *Gemmatimonadetes*^35,36^ were more represented. This might be because of the relatively higher SOC and TN in the soils with double-season straw and corn straw return. Wang *et al*.^37^ reported that the relative increase in SOC and TN was significantly linearly related to straw-C and -N inputs and there was no significant difference on the conversion of straw-C to SOC among different straw types. In this study, compared with wheat straw (~4900 and ~9800 kg ha^−1^), greater amount of corn straw (~5500 and ~11000 kg ha^−1^) was returned to the field during the experiment. Thus, it can be speculated that more straw derived-C and -N entered soil C and N pool and this was main reason for higher SOC and TN content in the treatment with corn straw return. However, the lower SOC contents in C-100% and W-50% than those in Blank and CW-0 were also observed in the study. It might be because the mineralization of soil organic matter was accelerated by straw return in these treatments, leading to positive priming effect for soils. The relative abundance of several bacterial phyla reduced after long-term of straw return, such as *Nitrospira* and *Planctomycetes*, which were mainly composed of oligotrophic species^35,38,39^. The decrease of the relative abundance of *Nitrospira* might due to the N availability reduction after straw return^9^, such as in CW-100% and C-100%.

**Figure 6:**
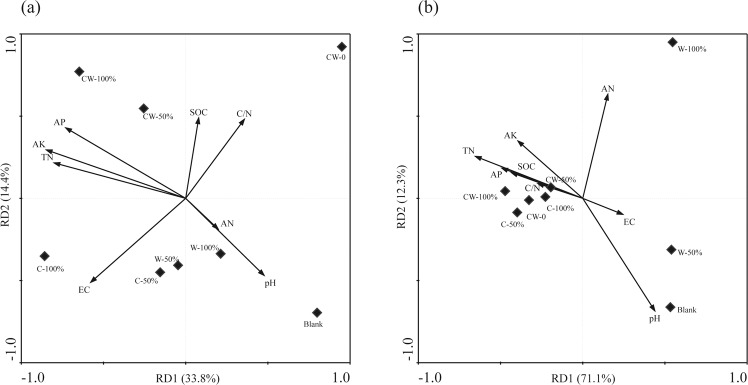
Redundancy analysis (RDA) for the correlation of bacterial (**a**) and fungal (**b**) community with the environmental factors, respectively.

RDA results showed that Blank and CW-0 were separated from the soils with straw return by Axis 1 (explaining 33.8% of total microbial community variation), more correlated with TN, AK and AP, while the soils with double-season straw return were separated from the soils with single-season straw return by Axis 2 (explaining 14.4% of total microbial community variation), more correlated with SOC, AN, C/N and pH (Fig. 6a). Among these factors, soil AK (*P* = 0.008) and C/N (*P* = 0.048) had significant impact on the soil bacterial community variations. It reported that during the process of straw decomposition, about 35–40% of straw-C can be converted to different types of SOC^40,41^, such as dissolved organic C and microbial biomass C, which can be used by soil microbes as energy and nutrient sources and then shift soil microbial community composition. Similarly, during the process of straw-C conversion, the elements of N and K in straws could be also transformed to the soil and further affect the microbial community as nutrients.

For soil fungal communities, the diversity indices decreased significantly in treatments with corn straw return (CW-50%, CW-100%, C-50% and C-100%). This was mainly due to the fact that certain species of fungi gradually became dominant after corn straw return. In the study, the genus of *Leptosphaeria*, with the relative abundance of 44.1%, 37.4%, 49.3% and 45.8% in CW-50%, CW-100%, C-50% and C-100%, respectively, might be induced to be the dominant with corn straw return. It reported that *Leptosphaeria* always exhibited in pathogen of wheat and disseminate by seed borne transmission^33,42^. Thus, it is speculated that corn straw return in October might increase the relative abundance of *Leptosphaeria*, which brought higher pathogenic risk for wheat. It reported that corn straw was more difficult to be decomposed than wheat straw under temperature of 1.5–17.6 °C^17^. Large amount of fresh corn straw might provide favorable temperature and water content for soil-borne pathogen growth during winter. But the further study on the *Leptosphaeria* diversity at the species level and on its abundance and activity is needed, to better reveal the effect of long-term corn straw return on health of soils and crops. In addition, the increase of *Oidiodendron* was only observed in the treatment with corn straw return, especially in CW-100%. It has been demonstrated that the colonization of *Oidiodendron* improves plant absorption of NO_3_^−^ under acidic growing conditions^43^. Thus, an increase in *Oidiodendron* might be associated with the higher soil TN after more straws return. Although the fungal community in Blank was more similar to that in soils with only wheat straw return, the community diversity increased slightly with only wheat straw return, and the abundance of *Mortierella* increased up to 20.8% and 41.3% in W-50% and W-100%, respectively. Their ability to produce chitinolytic enzymes made *Mortierella* successful competitors among other fungi in decomposing wheat straws^44^. This was in agreement with other reports that *Mortierella* increased to 20–30% in the cropland and grassland soils with wheat straw incorporation^9^.

RDA results indicated that soil EC (*P* = 0.04) were significant factors influencing the fungal community variations (Fig. 6b). Soil EC was more related to the compounds released from the straw decomposition such as organic acid, which then affected the salt compounds. It reported that the fresh corn straw during decomposition had higher C/N, O-alkyl/alkyl ratio and aromaticity than that of wheat straws^17^, which might be the reason for the different fungi selected to be the dominant by these two straws.

### Long-term straw return impacted soil microbial catabolic activity

The MicroResp provided a measure for the potential carbon utilization activities of heterotrophic bacteria. Most of the substrates selected in the study are the important components in root exudates, which could reflect the resistance to biotic and abiotic pressure of crops and indicators of crop disease^45^. Compared with other soils, significantly higher respiratory activity was observed in CW-100%, especially induced by L-cysteine, citric acid, oxalic acid and trehalose, which was probably due to the certain microbial groups adapted to degrading such substrates^46^. Oxalic acid was one common organic acid released from fungi, in order to form the sparingly soluble oxalate, including Ca, P and Mn oxalate, and then to support the growth of itself as well as the crops^47^. A significantly positive correlation was observed between the respiratory activities induced by oxalic acid and the fungi PLFA profiles in the soils (R = 0.726, *P* = 0.041), indicating that fungi increased by straw return was not only benefit for decomposing the straw, but also for releasing more available nutrient. This result was consistent with higher nutrients availability of the soils with straw return.

It has been demonstrated that many plants are capable to produce phytocystatins to inhibit the activity of cysteine proteinases, and then destroy the growth of plant pathogenic microbes^48^. Citric acid could be produced by plant to inhibit the microbial pathogens^49^. Trehalose is reported to be as an inducer of plant defense response^50^. The study results from Brodmann et al. suggested that pathogen can produce trehalose and use it as virulence tools for plants^51^. Thus, significantly higher respiratory rates induced by L-cysteine, citric acid and trehalose in CW-100% reflected a higher plant pathogen potential there. It was consistent with the result that much higher actinomycetes was observed in CW-100%, because actinomycetes have certain capacity to moderate the pathogenic effect by inhibiting the activity of pathogenic fungi^31,32^. Significantly positive correlations between the respiratory activities induced by trehalose, L-cystenine, citric acid and the abundance of actionomycete PLFA profiles (R = 0.964, 0.996, 0.994, *P* < 0.001) could also demonstrate the above speculations.

## Conclusion

Soil microbial properties varied with the type of returned straws and the return rates. In the study field, the microbial cell synthesis and catabolic activity increased more greatly with double-season straw return than single-season straw return, mainly for higher SOC, TN, AK and AP contents. The general soil microbial community structure varied with different straw return rates. Compared with single-season straw return, lower relative abundance of bacteria and fungi, but higher relative abundance of actinomycetes were observed in double-season straw return. Copiotrophic bacteria was more represented in the soil with corn straw return, while oligotrophic group was more represented in the soil with wheat straw return. Different functioning fungi was selected to be dominant by wheat and corn straws, identified to be *Mortierella* and unclassified *Ascomycota*, respectively. Compared with wheat straw return, lower fungal community diversity and higher fungal pathogenic risk, mainly caused by *Leptosphaeria*, were observed in the soil with corn straw return, especially at high return rates. Our study revealed that long-term of full corn straw return might increase the pathogenic risk for soils and further for crops. How to enhance the *in-situ* decomposition of corn straw during the wheat-season needs to be further studied in future.

## Supplementary information

Supplementary Information.

## Data Availability

Sequence data that support the findings of this study have been deposited in the Sequence Read Archive at the NCBI under the accession number of SRR5557210 under the Project ID of PRJNA384847. The data supporting the findings of soil physiochemical property and soil PLFA profile abundance of this study are available within the article and its supplementary information files.
