# Supplementary material for: Soil microbial community shifts with long-term of different straw return in wheat-corn rotation system
Source: Sci Rep. 2020 Apr 14;10:6360. doi: 10.1038/s41598-020-63409-6 (PMC7156462; doi:10.1038/s41598-020-63409-6)
Supplement: Supplementary file 1 — Supplementary Information. [file 41598_2020_63409_MOESM1_ESM.pdf]

Supplementary Materials

Soil microbial community shifts with long-term of different straw return in wheat-corn rotation system

Yao Su[1], Man Yu[1], Hui Xi[1], Jinling Lv[2], Zhenghua Ma[2], Changlin Kou[2], Alin Shen[1*]

[1]*Institute of Environment, Resource, Soil and Fertilizer, Zhejiang Academy of Agricultural Sciences, Hangzhou 310021, China*

[2]*Institute of Plant nutrient, Environment and Resource, Henan Academy of Agricultural Sciences, Zhengzhou 450002, China*

[*] Corresponding author. Tel.:+86 571 88166275; Fax: +86 571 88166275;

Email: shenalin_aee@163.com

Table S1 General description of soil properties of different straw return treatment. Different letters indicate significant difference (*P* < 0.05) analyzed by using ANOVA.

Treatment	pH	SOC (g kg^{-1})	TN (g kg^{-1})	C/N	AN (mg kg^{-1})	AK (mg kg-$^{-1}$)	AP (mg kg^{-1})	EC (μs cm^{-1})
Blank	8.90±0.12^{a}	8.92±0.62^{a}	0.84±0.01^{ab}	10.57±0.83^{a}	25.02±6.69^{ab}	129.5±2.12^{a}	3.15±1.65^{a}	215.0±3.4^{a}
CW-0	8.25±0.08^{b}	9.10±0.43^{a}	0.83±0.08^{a}	10.98±1.52^{ab}	38.54±4.78^{c}	126.5±0.71^{a}	6.5±0.45^{b}	119.8±5.2^{b}
CW-50%	8.20±0.05^{b}	11.39± 0.56^{b}	0.92±0.01^{c}	12.36±0.75^{a}	40.57±1.91^{c}	242.5±6.36^{b}	13.53±0.89^{c}	204.0±6.1^{a}
CW-100%	8.29±0.10^{b}	10.39±0.28^{c}	0.95±0.00^{c}	10.96±0.30^{b}	22.31±2.87^{a}	255.5±3.54^{c}	11.33±0.74^{d}	218.0±1.9^{a}
C-50%	8.31±0.12^{b}	10.28±0.40^{c}	0.92±0.03^{c}	11.14±0.76^{c}	60.86±9.56^{d}	178.0±2.83^{d}	8.47±0.10^{e}	200.0±1.6^{a}
C-100%	8.24±0.08^{b}	6.95±0.10^{d}	0.91±0.01^{cd}	7.62±0.21^{ad}	27.05±3.83^{ae}	194.0±1.41^{e}	9.36±0.10^{e}	200.2±2.5^{a}
W-50%	8.31±0.10^{b}	7.13±0.61^{d}	0.84±0.01^{a}	8.53±0.84^{a}	35.16±19.13^{bce}	171.0±1.41^{f}	11.23±0.10^{d}	198.2±1.2^{a}
W-100%	8.20±0.05^{b}	9.00±0.02^{a}	0.88±0.03^{bd}	10.28±0.28^{bd}	71.00±4.78^{d}	181.5±2.12^{d}	5.92±0.10^{b}	194.4±1.9^{a}

Table S2 The abundance of detected PLFA profiles in all treatments.

Microbial Community	PLFA profile	PLFA amount (nmol g_{soil}^{-1})							
		Blank	CW-0	CW-50%	CW-100%	C-50%	C-100%	W-50%	W-100%
Bacterial	15:0 iso	35.10	38.21	42.30	71.18	41.11	73.28	45.97	44.03
	15:0 anteiso	19.55	22.73	24.04	63.74	22.87	51.90	27.24	25.56
	16:0 iso	14.86	16.15	17.83	28.70	17.70	33.13	23.80	18.33
	16:0 anteiso	0.00	0.00	0.00	0.00	0.00	0.00	0.00	0.00
	16:1 w9c	0.00	0.00	0.00	0.00	0.00	12.73	0.00	0.00
	16:1 w11c	0.00	0.00	0.00	13.66	8.23	0.00	8.63	0.00
	16:1 w7c/16:1	42.92	45.99	48.98	84.22	41.53	95.52	54.37	86.78
	16:1 w5c	75.02	29.10	26.63	65.12	45.45	71.88	54.15	40.25
	17:1 anteiso	0.00	0.00	0.00	0.00	0.00	0.00	14.39	0.00
	17:0 iso	12.25	14.13	15.35	23.51	14.78	23.26	17.29	17.12
	17:0 anteiso	10.26	12.47	12.26	21.20	14.80	23.82	15.73	14.40
	17:1 w8c	0.00	0.00	3.54	7.71	7.24	12.61	4.17	6.98
	17:0 cyclo	11.03	12.44	14.27	20.19	16.29	31.53	14.65	11.68
	18:1 w7c	54.36	48.34	53.26	103.66	79.77	126.64	68.52	67.87
	18:1 w5c	0.00	0.00	4.76	0.00	0.00	0.00	3.17	15.46
	19:0 cyclo w8c	27.12	25.03	27.35	47.95	38.21	42.17	38.67	26.20
Actinomycetes	16:0 10-methyl	42.28	46.66	43.72	78.66	49.86	70.23	63.96	52.31
	17:0 10-methyl	0.00	5.82	5.47	7.63	7.04	12.54	4.87	3.80
	18:0 10-methyl,	10.82	13.65	13.11	17.86	13.04	17.56	15.85	14.33
	19:0 10-methyl	0.00	0.00	0.00	962.92	0.00	0.00	0.00	0.00
Fungal	18:2 w6,9c/18:0	10.33	18.45	24.27	84.94	38.49	72.33	35.20	32.55
	18:1 w9c	43.61	50.45	53.60	160.04	40.62	134.82	76.08	75.79
Others	9:00	2.69	0.00	0.00	0.00	2.60	0.00	0.00	0.00
	10:00	3.13	0.00	1.61	3.86	2.23	0.00	0.00	0.00
	12:00	0.00	5.11	5.37	0.00	5.32	4.96	5.88	0.00
	14:0 iso	0.00	4.42	4.89	8.43	0.00	8.04	5.17	3.88
	14:00	0.00	9.67	14.05	23.77	0.00	10.18	14.82	9.10
	15:1 iso G	0.00	0.00	2.54	0.00	3.68	7.12	0.00	2.38
	15:1 anteiso A	0.00	0.00	0.00	0.00	0.00	3.42	0.00	0.00
	15:00	2.11	2.91	4.99	877.67	2.55	9.87	6.43	5.71
	16:00	96.29	137.58	158.73	230.18	115.92	291.02	174.59	178.12
	17:00	0.00	3.24	18.89	0.00	0.00	11.35	6.16	4.83
	16:1 2OH	0.00	0.00	24.60	26.54	0.00	10.88	16.90	9.33
	16:0 2OH	9.58	0.00	3.27	0.00	11.18	0.00	4.44	5.25
	18:00	27.89	68.86	92.91	96.71	66.16	124.25	91.19	97.73
	18:1 w7c	0.00	0.00	0.89	5.81	0.00	10.47	2.54	6.48
	19:00	50.00	50.00	50.00	50.00	50.00	50.00	50.00	50.00
	20:4	0.00	0.00	0.00	0.00	0.00	9.10	0.00	4.92
	20:00	0.00	8.15	6.83	11.26	0.00	24.98	8.80	11.61
Total		601.19	689.56	820.30	3197.15	756.68	1481.60	973.62	942.81

Table S3 Catabolic profiling obtained with MicroResp™ assay in response to the different treatments.

Carbon source		Substrates induced respiratory rates (μg CO_2 g_{soil}^{-1} h^{-1})							
		Blank	CW-0	CW-50%	CW-100%	C-50%	C-100%	W-50%	W-100%
Water		0.65	2.14	1.46	5.00	0.46	0.86	0.46	0.88
carbohydrates	D-Glucose	0.46	1.38	1.82	4.99	1.56	2.12	1.90	1.84
	L-Arabinose	0.66	1.71	1.85	9.14	0.36	0.38	0.38	0.74
	D-Fructose	1.03	2.94	4.18	4.84	0.89	0.90	1.03	1.43
	D-Galactose	0.49	2.41	2.59	2.91	0.36	0.44	0.32	0.61
	Trehalose	0.53	2.21	2.48	10.72	0.31	0.42	0.41	0.75
Amino acids	L-Alanine	0.40	2.22	2.09	4.21	0.33	0.27	0.25	0.56
	Arginine	4.49	3.34	4.52	10.37	3.27	2.42	3.39	3.89
	L-Cysteine HCl	5.59	3.33	5.76	47.52	5.01	4.07	5.03	5.69
	L-Lysine	0.62	2.35	2.59	4.41	0.43	0.45	0.45	0.45
	NAGA	4.29	3.61	3.82	1.31	3.70	4.35	4.91	5.54
Organic acids	Citric acid	5.49	2.87	5.15	30.35	3.87	4.88	4.43	5.32
	GABA	0.74	3.23	3.16	7.09	0.60	0.60	0.52	0.73
	L-Malic acid	0.01	0.03	0.39	0.94	0.00	0.00	0.00	0.00
	3,4-OHbenzoic acid	0.52	2.64	3.11	6.52	0.46	0.77	0.57	0.74
	Oxalic acid	2.40	3.44	3.85	38.03	1.62	2.62	2.08	2.19
